# Circular RNA circMET drives immunosuppression and anti-PD1 therapy resistance in hepatocellular carcinoma via the miR-30-5p/snail/DPP4 axis

**DOI:** 10.1186/s12943-020-01213-6

**Published:** 2020-05-19

**Authors:** Xiao-Yong Huang, Peng-Fei Zhang, Chuan-Yuan Wei, Rui Peng, Jia-Cheng Lu, Chao Gao, Jia-Bing Cai, Xuan Yang, Jia Fan, Ai-Wu Ke, Jian Zhou, Guo-Ming Shi

**Affiliations:** 1 Department of Liver Surgery and Transplantation, Liver Cancer Institute, Zhongshan Hospital, Fudan University, Key Laboratory of Carcinogenesis and Cancer Invasion, Ministry of Education, Fudan University, 180 Fenglin Road, Shanghai, 200032 P.R. China; 2 Cancer Center, Institutes of Biomedical Sciences, Fudan University, Shanghai, 200031 P.R. China; 3 State Key Laboratory of Genetic Engineering and Collaborative Innovation Center for Genetics and Development, School of Life Sciences, Fudan University, Shanghai, China; 4 Shanghai Key Laboratory of Organ Transplantation, Zhongshan Hospital, Fudan University, Shanghai, China

**Keywords:** Hepatocellular carcinogenesis, circRNA, miR-30-5p, circMET, DPP4

## Abstract

**Background:**

Amplification of chromosome 7q21-7q31 is associated with tumor recurrence and multidrug resistance, and several genes in this region are powerful drivers of hepatocellular carcinoma (HCC). We aimed to investigate the key circular RNAs (circRNAs) in this region that regulate the initiation and development of HCC.

**Methods:**

We used qRT-PCR to assess the expression of 43 putative circRNAs in this chromosomal region in human HCC and matched nontumor tissues. In addition, we used cultured HCC cells to modify circRNA expression and assessed the effects in several cell-based assays as well as gene expression analyses via RNA-seq. Modified cells were implanted into immunocompetent mice to assess the effects on tumor development. We performed additional experiments to determine the mechanism of action of these effects.

**Results:**

circMET (hsa_circ_0082002) was overexpressed in HCC tumors, and circMET expression was associated with survival and recurrence in HCC patients. By modifying the expression of circMET in HCC cells in vitro, we found that circMET overexpression promoted HCC development by inducing an epithelial to mesenchymal transition and enhancing the immunosuppressive tumor microenvironment. Mechanistically, circMET induced this microenvironment through the miR-30-5p/Snail/ dipeptidyl peptidase 4(DPP4)/CXCL10 axis. In addition, the combination of the DPP4 inhibitor sitagliptin and anti-PD1 antibody improved antitumor immunity in immunocompetent mice. Clinically, HCC tissues from diabetic patients receiving sitagliptin showed higher CD8^+^ T cell infiltration than those from HCC patients with diabetes without sitagliptin treatment.

**Conclusions:**

circMET is an onco-circRNA that induces HCC development and immune tolerance via the Snail/DPP4/CXCL10 axis. Furthermore, sitagliptin may enhance the efficacy of anti-PD1 therapy in a subgroup of patients with HCC.

## Introduction

Hepatocellular carcinoma (HCC) is the most frequent hepatic malignancy and has a poor prognosis [1]. To date, the only option for curative treatment for HCC is surgical resection or liver transplantation, but most patients are usually diagnosed at an advanced stage and are not candidates for surgery. Moreover, tumors commonly reappear after surgery, and the 5-year postoperative survival rate is poor [2]. Therefore, a deeper investigation of the mechanisms associated with HCC progression and recurrence is of clinical significance and can lead to the development of new therapeutic approaches for patients with HCC [3].

The discovery of the constitutive activation of the mitogen-activated protein kinase (MAPK) pathway as a result of gene mutation or amplification in cancer has revolutionized traditional cancer medicine by enabling precision medicine for a subgroup of patients [4]. Furthermore, the emergence of cancer immunosurveillance introduced immune checkpoint blockades to cancer therapy [5]. However, the majority of patients treated with these agents show a minimal clinical response and sometimes develop drug resistance, which leads to further disease development and death [6]. Thus, the molecular mechanisms of HCC are still being studied.

Many molecular events have been linked to the development and progression of HCC, such as overactivation of *c-Myc* and inactivation of *Pten* and *p53* [7]. Over the past few decades, significant advances have revealed chromosome aberrations in HCC, including gains of 1q21–23 and 8q22–24, that are involved in the early development of HCC, and the gain of 3q22–24 has been associated with tumor recurrence and poor survival [8]. Chromosomal amplification at 7q21-7q31 was reported to be closely related to tumor recurrence [9–12], and several oncogenes, such as *MET* and *PEG10*, in this region are amplified in many cancers [13, 14]. Thus, specific chromosomal aberrations can be responsible for carcinogenesis via dysregulation of tumor suppressor genes or oncogenes. Considering the hundreds of genes in a chromosomal region, more work is needed to reveal additional tumor-related genes in regions with chromosomal aberrations [15].

Circular RNAs (circRNAs), a novel kind of regulatory RNA characterized by a continuous covalent closed loop without a 5′-cap structure or 3′-poly A tail, are considered splicing error byproducts. Hundreds of circRNAs exist in mammalian cells and regulate a broad range of biological processes through various mechanisms, including sponging of microRNAs (miRNAs) [16]. Aberrant expression of circRNA has also been implicated in the initiation and development of various diseases, including cancers [17]. For example, circTRIM33–12 acts as a sponge of miRNA-191 to inhibit HCC progression [18], while circHIPK3 is a tumor suppressor in bladder cancer [19]. As circRNAs are novel endogenous noncoding RNAs, the biological functions of most circRNAs and their underlying mechanisms in the pathogenesis of HCC remain largely unclear and need further exploration [20].

Most cells cannot survive an attack from the immune system, with the exception of tumor cells due to their loss of immunogenicity and immunosuppression at malignant mass sites [21]. Evading immune destruction has been deemed an emerging hallmark of cancer, and the crosstalk between the immune system and tumor cells plays a definitive role in progression to advanced stages of cancer [22]. Moreover, the host immune system can combat cancers and improve outcomes for cancer patients, which has been documented by clinical testing of immune checkpoint blockades [23]. A programmed cell death 1 (PD1) antibody has been approved for second-line therapy in advanced HCC [24]. However, only 17–18% of patients with advanced HCC have achieved complete or partial response to anti-PD1 antibody therapy, and adverse events have been observed, indicating that a better understanding of cancer immunosuppression is needed [25].

Previous work has implicated chr.7q21-7q31 amplification in the progression of HCC [9, 26, 27]. This region contains critical oncogenes involved in tumor progression, such as MET proto-oncogene receptor tyrosine kinase (*MET*)*,* hepatocyte growth factor (*HGF*)*,* SEM1 26S proteasome complex subunit (*SHFM1*)*,* minichromosome maintenance complex component 7 (*MCM7*), and ATP binding cassette subfamily B member 4 (*ABCB4*, 12]. Therefore, we speculated that chr.7q21-7q31-derived circRNAs could act as oncogenes or tumor suppressors in HCC. Here, we determined the expression of circRNAs in the 7q21-7q31 region in human HCC tissues and correlated this expression with HCC patient survival and disease recurrence. We modified the expression of the identified circRNAs to determine whether they can promote HCC development in HCC cells in vitro. Modified cells were also implanted into immunocompetent mice to assess the effects on tumor development. Then, we performed additional experiments to determine the mechanism of action of these effects.

## Methods

### Cell lines, animals and transfection of lentiviral vectors

We used the human HCC cell lines HCCLM3 (Liver Cancer Institute, Zhongshan Hospital), and Huh7, Hep3B, and HepG2 (American Type Culture Collection) with high/low metastatic capacity and the murine HCC cell line Hep1–6 (American Type Culture Collection). Cells were grown at 37 °C and 5% CO_2_.

C57BL/6 mice were cultured in specific pathogen-free conditions (Shanghai Institute of Material Medicine, Chinese Academy of Science). Animal care and experimental protocols were in line with the guidelines of the Shanghai Medical Experimental Animal Care Commission.

Lentiviral vectors containing circMET, miR-30a-5p, snail, shcircMET, shmiR-30a/e-5p, simiR-30b-5p, simiR-30c-5p, or simiR-30d-5p were constructed (Shanghai Genomeditech Co. Ltd., Shanghai, China). Stable transfectants were characterized by quantitative real-time polymerase chain reaction (qRT-PCR) or western blotting. The targets of sh/si-miR-30-5p are listed in Additional file 1**: Table S1.**

### Patients and follow-up

Specimens obtained from the vicinity of the tumor margin were collected from 209 patients with HCC who underwent radical resection at the Fudan University Liver Cancer Institute (Shanghai, China) from 2006 to 2008. Ethical approval was confirmed by the Zhongshan Hospital Research Ethics Committee, and written informed consent was obtained from each patient. Follow-up data were ended by March 2014, and the follow-up median time was 62 months (range 4 ~ 121 months).

### circRNA immunoprecipitation (circRIP) and in situ hybridization

circRNAs precipitation in vivo, circRNAs immunoprecipitation and in situ hybridization were performed as previously described with minor modifications, and the procedures are described in the Additional file 4: supplementary materials and methods [18, 28].

### Quantitative real-time polymerase chain reaction analysis, western blotting analysis, immunofluorescence assays, 3-(4,5-dimethylthiazol-2-yl)-2,5-diphenyltetrazolium dromide assay, cell migration, Matrigel invasion assays, and gene microarray

These experiments were performed according to previous reports [14, 18, 29], and the procedures are described in the Additional file 4: supplementary materials and methods.

### Human CD8^+^ T cell isolation and preparation and CD8 T cell chemotaxis assay

Blood was collected from HCC patients to isolate peripheral blood mononuclear cells using Ficoll. Then, CD8^+^ T cells were purified using anti-CD8 antibody-coupled immunomagnetic beads and resuspended in RPMI 1640 in the supplemented with 2% FBS. CD8^+^ T lymphocyte chemotaxis was assayed in a Transwell system (Corning, Tewksbury, MA, USA) using 5-μm polycarbonate membranes, as described previously with modifications [30]. Human CXCL10 (50 ng/ml) was added to RPMI 1640 supplemented with 2% FBS and incubated with HCC cells for 24 h, after which the supernatant was added to the bottom wells. Then, CD8^+^ T lymphocytes from the peripheral blood of HCC patients suspended in RPMI 1640 with 2% FBS were added to the top wells and incubated 2 h. Following incubation, CD8^+^ T lymphocytes that migrated to the lower chamber were counted using a cell counter. The chemotactic index was evaluated as the ratio of the number of CD8^+^ T lymphocytes that migrated to CXCL10-containing supernatant wells divided by the number of CD8^+^ T lymphocytes that migrated to RPMI 1640 alone.

### Immunohistochemistry (IHC)

Immunohistochemical staining of the target circRNAs and proteins was carried out on a tissue microarray (TMA). The circMET expression was quantified as follows: three images of representative fields of circMET staining were captured under a computerized image system. Images were analyzed with Image-Pro Plus version 6.2 software (Media Cybernetics) using a special function called measurement of integrated absorbance, which evaluates both the area and the intensity of the positive staining. The density of circMET (the integrated optical density of all positive staining/total area) was calculated as representative of a particular sample, and the mean density was determined and used as a cutoff in subsequent analyses. The quantification of dipeptidyl peptidase 4(DPP4) was also performed via Image-Pro Plus version 6.2 software. For the CD8 T infiltrating lymphocytes in the TMA, positive cells in 200× images were counted in each section. For snail staining, the area of positive staining in an image was measured, and the average proportion (area of positive staining/total area) for each spot was calculated to represent a particular sample. The cutoff value was the mean of the data. The cutoff value for snail was 28% and the cutoff number of CD8^+^ tumor infiltrating lymphocytes was 30 in the 200× images.

### Chromatin immunoprecipitation sequencing (CHIP-seq)

DNA from HCC cells was sonicated into 200–800 bp fragments and precipitated by centrifugation. DNA was resuspended in PBS and incubated with anti-Snail antibody (2–5 μg for 25 μg DNA) at 37 °C for 4 h. Samples were incubated with prewashed magnetic beads, collected with a magnetic frame, and washed with PBS. DNA was extracted with phenol chloroform, precipitated, and concentrated to prepare ChIP samples. DNA detection was performed by RT-PCR. Samples were constructed according to the Illumina library protocol and sequenced using the Illumina sequencing system.

### Dual luciferase reporter assay

A mutant luciferase reporter vector was generated using a mutagenesis kit (Qiagen, CA, USA) according to the manufacturer’s instructions. Plasmids were transiently transfected into 293 T cells, which were lysed and collected after 48 h. Samples were centrifuged at 10,000–15,000 rpm for 3–5 min, and supernatants were collected.

Luciferase detection was performed according to the instrument instructions with a measurement time of 10 s and an interval of 2 s. For the assay, 20 μL of sample and 20 μL of firefly luciferase assay reagent were gently mixed 2–3 times, and relative light units (RLU) were measured with cell lysis buffer as a blank control. This process was repeated with Renilla luciferase assay reagent, and the degree of reporter gene activation was determined by using the ratio of both RLU values.

### Chemokine chip

A chemokine chip assay was performed via a Raybiotech mouse cytokine array and the steps are listed in Additional file 4: supplementary materials and methods.

### In vivo tumor growth and metastasis assays

The in vivo tumor growth assays were performed using C57BL/6 mice. Mice were acquired from the Shanghai Institute of Material Medicine and were fed in a pathogen-free environment. Hep1–6 cells were injected subcutaneously into the mice with a 27-gauge needle. Tumor sizes were calculated by the following formula: volume (mm^3^) = [width^2^ (mm^2^) × length (mm)]/2.

### Statistical analysis

Statistical analysis was performed with SPSS software (19.0, SPSS, Inc., Chicago, IL) as previously described [4]. Values are shown as the mean ± standard deviation. The chi-square test and Fisher’s exact probability test were used for comparisons between groups. Correlation analysis was performed between circMET and Met. The recurrence and overall survival (OS) in HCC patients were analyzed using Kaplan-Meier’s method and the log-rank test. Cox’s proportional hazards regression model was employed to present the independent prognostic factors. *p* < 0.05 was considered statistically significant.

## Results

### circMET was overexpressed in HCC tissues, and a high level of circMET was associated with poor patient survival

**Fig. 1:**
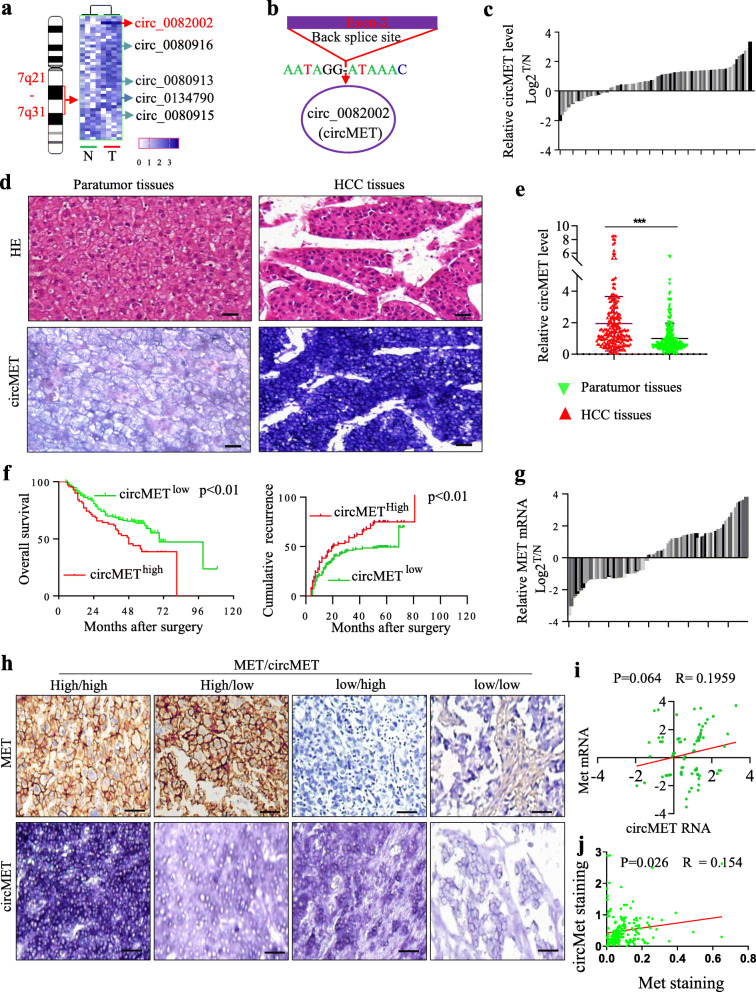
circMET overexpression is negatively related to poor survival in HCC patients **a**. circRNA expression in chr. 7q21-7q31 was determined by qRT-PCR in four pairs of HCC and matched adjacent nontumor liver tissues; **b**. Schematic illustration of the circularization of MET exon 2 forming circMET; **c**. The RNA expression of circMET was determined in 90 pairs of HCC and matched adjacent nontumor liver tissues; **d**. The expression of circMET was determined by in situ hybridization in 209 pairs of HCC and matched adjacent nontumor liver tissues (bar = 200 nm); **e**. The scatter diagram shows the expression of circMET determined by in situ hybridization in 209 pairs of HCC and matched adjacent nontumor liver tissues; **f**. circMET expression, analyzed by in situ hybridization in 209 HCC patients, is related to the overall survival and cumulative recurrence of HCC patients; **g**. The expression of MET was determined by qRT-PCR in 90 pairs of HCC and matched adjacent nontumor liver tissues; **h**. The expression of MET and circMET was determined by immunohistochemistry and in situ hybridization in 209 pairs of HCC and matched adjacent nontumor liver tissues (bar = 200 nm); **i**. No association was found between MET and circMET levels, as determined by qRT-PCR in 90 HCC tissues; **j**. A slight association was found between MET and circMET levels, as determined by IHC and in situ hybridization in HCC samples

We speculated that chr.7q21-7q31-derived circRNAs could act as oncogenes or tumor suppressors of HCC. To verify our hypothesis, we used qRT-PCR to analyze the expression of the 43 circRNAs from chr.7q21-7q31 in four pairs of HCC tissues and matched adjacent nontumor liver tissues. Among these circRNAs, the expression of circMET (hsa_circ_0082002, a 1214 bp circRNA) was the most significantly and consistently upregulated in HCC tissues compared to nontumor liver tissues (Fig. 1a, b).

To elucidate the clinical relevance of circMET in HCC patients, we analyzed the expression of circMET in 90 pairs of HCC samples and matched adjacent liver tissues using qRT-PCR (Fig. 1c) and in a TMA consisting of 209 HCC tissues using in situ hybridization (Fig. 1d, f). Expression of circMET was significantly increased in tumor tissues compared to paired adjacent nontumor tissues, and the expression was primarily localized in the cytoplasm. Furthermore, circMET overexpression was significantly correlated with microvascular invasion (*p* = 0.047), absent tumor encapsulation (*p* = 0.025), multiple tumors (*p* = 0.031) and late HCC stage (Liver Cancer Guidelines in China, 2017 [31]) (Table 1). The high circMET expression groups accounted for 34% (71/209) of the total number of patients.

**Table 1:** Correlations between circMET and clinicopathological features in 209 HCC

variables	circMET high	circMET low	*P* value
Age (years)			
≥ 52	33	60	0.168
< 52	38	78	
Sex			
Male	66	113	0.031
Female	5	25	
Liver cirrhosis			
Yes	63	124	< 0.802
No	8	14	
HBsAg			
Positive	62	111	0.212
Negative	9	27	
Tumor size (diameter, cm)			
≤ 5	35	82	0.163
> 5	36	56	
Tumor number			
Multiple	17	17	0.031
Single	54	121	
Microvascular invasion			
Yes	28	36	0.047
No	43	102	
Tumor encapsulation			
Yes	27	75	0.025
No	44	63	
HCC stage			
Ia	27	81	0.020
Ib	31	44	
IIa	12	13	
IIb	1	0	

The expression of circMET was also correlated with overall survival (OS) and cumulative recurrence rates **(**Fig. 1f). Furthermore, the 2-year and 5-year OS rates in the circMET^low^ group were significantly higher than those in the circMET^high^ group (81.0% versus 68.6, and 59.2% versus 38.8%, respectively). Likewise, the 2- and 5-year cumulative recurrence rates in the circMET^low^ group were significantly lower than those in the circMET^high^ group (40.8% versus 52.9, and 49.5% versus 74.9%, respectively, Fig. 1f). Multivariate analysis identified circMET expression as an independent predictor of OS and postoperative recurrence (Table 2). Moreover, the expression of circMET and c-Met showed a slight correlation **(**Fig. 1g-j**).** Together, these results indicate that elevated circMET expression may contribute to the progression of HCC.

**Table 2:** Univariate and multivariate analyses of factors associated with survival and recurrence

Factors	OS				Cumulative recurrence			
	Univariate, *P*	multivariate			Univariate, *P*	multivariate		
		HR	95% CI	*p* value		HR	95% CI	*p* value
Age (years) (< 52 vs. ≥52)	0.888			NA	0.469			NA
Sex (female vs. male)	0.060			NA	0.127			NA
HBsAg (positive vs. negative)	0.183			NA	0.622			NA
Liver cirrhosis (yes vs. no)	0.930			NA	0.127			NA
Tumor size (diameter, cm) (> 5 vs. ≤ 5)	0.001	1.728	1.148–2.600	0.009	< 0.001	1.729	1.194–2.502	0.004
Tumor encapsulation (absent vs. present)	0.195			NA	0.043			NS
microvascular invasion (yes vs. no)	< 0.001	2.365	1.536–3.640	< 0.001	< 0.001	1.664	1.119–2.476	0.012
Tumor number (multiple vs. single)	0.045	1.213	0.735–2.002	NS	0.002	1.587	1.005–2.507	0.048
HCC staging	0.002			NA	< 0.001			NA
CircMET expression (high vs. low)	0.007	1.522	1.010–2.294	0.045	0.004	1.480	1.023–2.142	0.037

Abbreviations and Note: *OS* overall survival, *NA* not adopted, *NS* not significant, *HBsAg* hepatitis B surface antigen, *95%CI* 95% confidence interval, *HR* Hazard ratio, Cox proportional hazards regression model

### circMET increases HCC cell invasion and metastasis

**Fig. 2:**
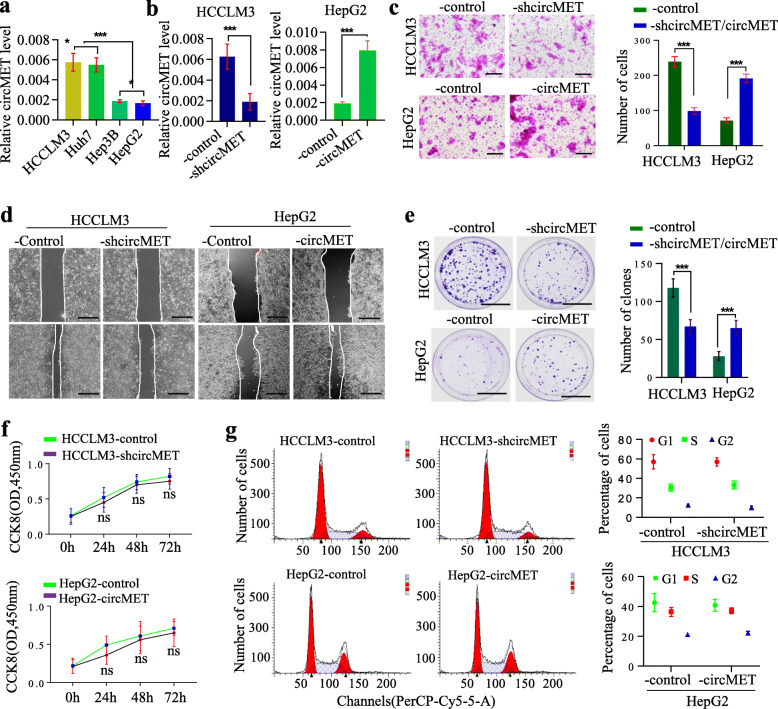
High levels of circMET promote HCC progression **a**. The expression of circMET in HCC cells was determined by qRT-PCR; **b**. The expression of circMET in HCCLM3 cells was inhibited, and in HepG2 cells, it was elevated; **c**. The invasion abilities of HCC cells with silenced or forced expression of circMET were evaluated by a transwell assay. Left, the representative pictures of transwell assay. Right, statistic of transwell assay; **d**. The migration abilities of HCC cells with silenced or forced expression of circMET were evaluated by the scratch assay; **e**. Colony formation assays were performed in the indicated cells. Left, representative pictures of colony formation assay. Right, statistic of colony formation assay; **f**. Proliferation in HCC cells with silenced or forced expression of circMET was assessed by MTT assay; **g**. The cell cycle in HCC cells with silenced or forced expression of circMET was assessed by FCM

To better understand the biological functions of circMET in HCC development, we examined the expression of circMET in HCCLM3, Huh7, Hep3B and HepG2 cells via qRT-PCR. The highly metastatic HCCLM3 cells expressed the highest levels of circMET, while the less metastatic HepG2 cells expressed the lowest levels of circMET **(**Fig. 2a). We then constructed a lentiviral vector for overexpressing circMET or a lentiviral vector containing shRNA-circMET to knockdown circMET to established stable cell lines (HCCLM3-shcircMET and HepG2-circMET)(Fig. 2b).

Transwell assays showed that invasive capacities were dramatically enhanced in HCC cells with high circMET levels compared to cells with low circMET levels (Fig. 2c). Wound healing assays showed delayed wound closure in HCCLM3-shcircMET and HepG2-control cells compared with HCCLM3-control and HepG2-circMET cells, respectively (Fig. 2d). In colony formation assays, ectopic expression of circMET enhanced colony formation, while inhibiting circMET reduced colony formation (Fig. 2e). In cell proliferation and cell cycle assays, circMET expression had no obvious impact on HCC cells (Fig. 2f, g). Taken together, these observations indicate that circMET is a positive regulator of HCC.

### Elevated circMET expression induces epithelial to mesenchymal transition and favors an immunosuppressive microenvironment in HCC

**Fig. 3:**
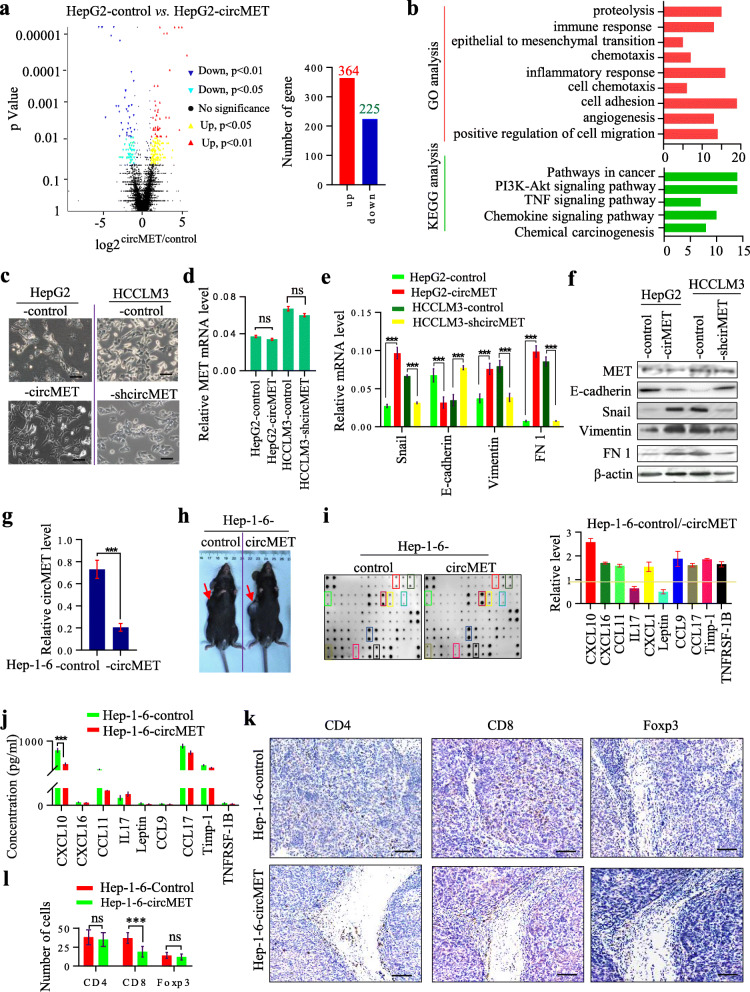
Establishments of murine and human HCC cells with EMT-like features and immunosuppression by circMET **a.** Volcano plots show the differentially expressed genes in HCC cells with different circMET expression levels; **b**. GO and KEGG analyses were performed on the differentially expressed genes; **c**. The cellular morphology of HCC cells with silenced or forced expression of circMET; **d**. The expression of MET was determined by qRT-PCR in HCC cells with silenced or forced expression of circMET; **e**. The expression of E-cadherin, vimentin, FN1 and Snail was determined by qRT-PCR in HCC cells with silenced or forced expression of circMET; **f**. The levels of E-cadherin, vimentin, FN1 and Snail protein were determined by western blotting in HCC cells with silenced or forced expression of circMET; **g**. circMET levels were elevated in Hep1–6 cells transfected with a circMET expression plasmid; **h**. Tumorigenesis of Hep1–6-control and Hep1–6-circMET cells in C57BL/6 mice, and the tumor burden of Hep1–6-circMET cells was larger than that of Hep1–6-control cells; **i**. Chemokine chips were used to determine the differences in chemokines between the sera of mice implanted with Hep1–6-control and Hep1–6-circMET cells; **j**. The top ten different chemokines between the sera of mice implanted with Hep1–6-control and Hep1–6-circMET cells were further assayed by ELISA; **k**. CD4^+^, CD8^+^ and Treg T cells were evaluated by immunohistochemistry in tumors derived from Hep1–6-control and Hep1–6-circMET cells; l. The histogram shows the CD4^+^, CD8^+^ and Treg T cells in tumors derived from Hep1–6-control cells and Hep1–6-circMET cells

To investigate the mechanism through which circMET promotes a metastatic profile in HCC, we conducted RNA-seq to identify differentially expressed genes between HepG2-control and HepG2-circMET cells. We identified 364 genes that were upregulated and 225 genes that were downregulated in HepG2-circMET cells compared to control cells (Fig. 3a**,** Additional File 5: Fig. S1a, Additional file 2: Table S2). Gene annotation enrichment analysis showed that differentially expressed genes were significantly enriched in pathways in cancer, chemokine signaling pathway, TNF signaling pathway and PI3K-Akt signaling pathway based on KEGG analysis, as well as epithelial to mesenchymal transition (EMT), cell adhesion, angiogenesis, cell chemotaxis, inflammatory response, chemotaxis and proteolysis based on GO analysis. (Fig. 3b).

Next, we further determined the functions of circMET in EMT and the tumor immune response. We analyzed the morphology of HCC cells expressing different levels of circMET. As shown in Fig. 3c, significant morphological differences were observed between HepG2-control or HCCLM3-mock cells and their corresponding cells with modified circMET expression. HepG2-control and HCCLM3-shcircMET cells exhibited the typical cobblestone-like appearance of normal epithelial cells, while HepG2-circMET and HCCLM3-control cells exhibited spindle-like fibroblastic morphology. However, no obvious difference in MET expression was detected between HepG2-control and HCCLM3-control cells and corresponding cells with modified circMET expression (Fig. 3d). HepG2-circMET cells expressed lower levels of the epithelial marker gene E-cadherin than HepG2-control cells. Compared to those in HepG2-control and HCCLM3-shcircMET cells, the levels of the transcription factor Snail and multiple mesenchymal genes [N-cadherin, fibronectin 1 (Fn 1) and vimentin] were increased in HepG2-circMET and HCCLM3 cells, respectively. In contrast, HCCLM3-control and HepG2-circMET cells expressed low levels of E-cadherin and high levels of N-cadherin, Fn1 and vimentin. **(**Fig. 3e, f**)**.

To determine the role of circMET in the tumor immune response, we also established stable Hep1–6-circMET and Hep1–6-control cells **(**Fig. 3g), and subcutaneously implanted these cell lines in C57BL/6 mice (Hep1–6-circMET cells and Hep1–6-control cells). Mice implanted with Hep1–6-circMET cells had a larger tumor burden than those implanted with Hep1–6-control cells (Fig. 3h). We then performed quantitative analyses of several cytokines and chemokines to compare serum cytokine profiles of C57BL/6 mice implanted with Hep1–6-circMET or Hep1–6-control cells using a liquid protein chip (Liqui Chip). Several cytokines and chemokines showed significant differences between the two groups, including CXCL10, CXCL16, CCL11, IL17, leptin, CCL9, CCL17, Timp-1 and TNFRSF1B (Fig. 3i), which was further confirmed by ELISA (Fig. 3j).

Importantly, in vivo experiments showed that the density of tumor-infiltrating CD8^+^ lymphocytes was significantly higher in tumors derived from mice injected with Hep1–6-control cells than Hep1–6-circMET cells (Fig. 3k, l). Additionally, clinical data showed that high levels of circMET were significantly correlated with a low density of tumor-infiltrating CD8^+^ lymphocytes in HCC tissues (Additional files 5**:**Fig. S1b), and a low density of intratumoral cells predicted poor prognosis in HCC patients. (Additional files 5:Fig. S1c). Therefore, we suggest that circMET promotes HCC development by inducing cellular EMT and favoring an immunosuppressive tumor microenvironment.

### circMET acts as a sponge for the miR-30-5p family

**Fig. 4:**
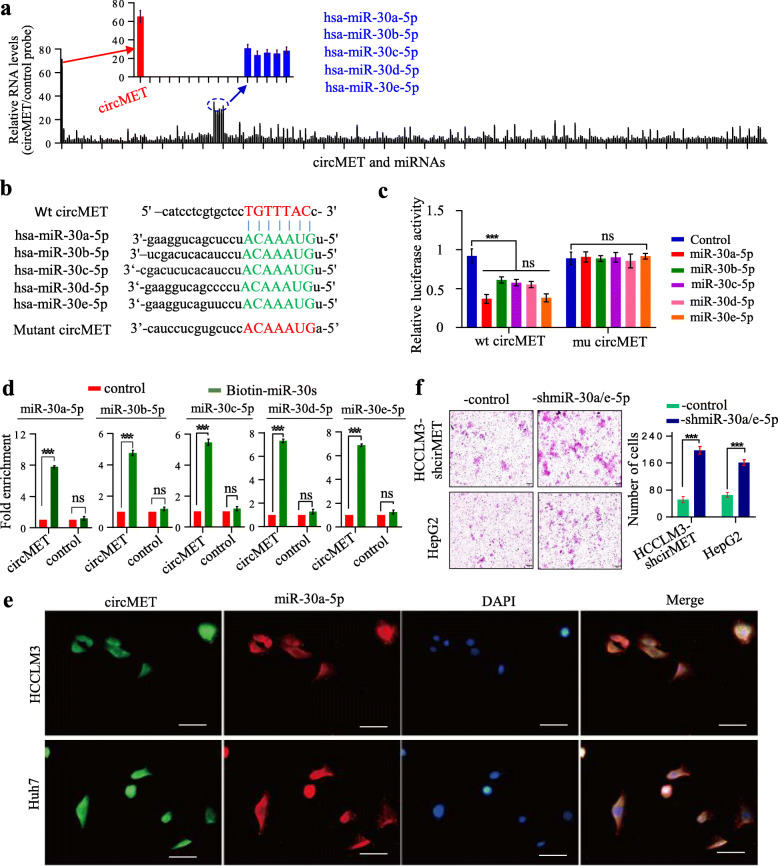
circMET may function as a sponge for miR-30-5p **a**. The circRIP assay showed a specific enrichment of circMET and miR-30-5p compared with that in the negative control; **b**. A schematic drawing showing the putative binding sites of wt-miR-30s-5p and mu-miR-30s-5p in circMET; **c**. The luciferase activity of luc-circMET and mutant luc-circMET in HEK293T cells after cotransfection with miR-30-5p; **d**. The circRIP assay was performed in HEK293T cells using biotin-labeled miR-30-5p mimics and a negative control (NC); **e**. FISH analysis in HCCLM3 and Huh7 cells revealed that circMET colocalized with miR-30a-5p in the cytoplasm; **f**. miR-30a/e-5p knockdown in cells with low levels of circMET upregulated the cell invasive ability. Left, representative pictures of transwell assay. Right, statistic of transwell assay

Considering that circRNAs have been reported to act as miRNA sponges, we assessed whether circMET could sponge certain miRNAs to affect HCC progression [32]. To that end, we purified circMET-interacting miRNAs, by circRIP, using circMET-specific probes in HCCLM3 cells, and identified 385 candidate miRNAs (predicted by StarBase v3.0) using qRT-PCR. The results showed a specific enrichment of miR-30-5p (miR-30a-5p, miR-30b-5p, miR-30c-5p, miR-30d-5p and miR-30e-5p) with circMET compared to the negative control (Fig. 4a).

To further verify that miR-30-5p is sponged by circMET, we performed a dual-luciferase reporter assay in HEK293T cells. Full-length wild type (WT) circMET and mutant circMET without miR-30-5p binding sites were cloned into the luciferase reporter vector pLG3 (Fig. 4b). A miR-30-5p mimic significantly reduced the luciferase activity of the WT-circMET but not mutant-circMET reporter (Fig. 4c). Furthermore, a pull-down assay with a biotinylated miR-30-5p mimic showed significant enrichment of circMET compared to the negative controls (Fig. 4d).

In addition, FISH analysis in HCCLM3 and Huh7 cells showed that circMET colocalized with miR-30a-5p in the cytoplasm (Fig. 4e). In the transwell assay, decreased expression of miR-30a/e-5p in HCC cells with high levels of circMET resulted in enhanced invasion ability in vitro (Fig. 4f). Thus, circMET acts as a sponge for miR-30-5p to promote HCC progression.

### circMET induces immunosuppression in HCC through the miR-30-5p/snail/DPP4 axis

**Fig. 5:**
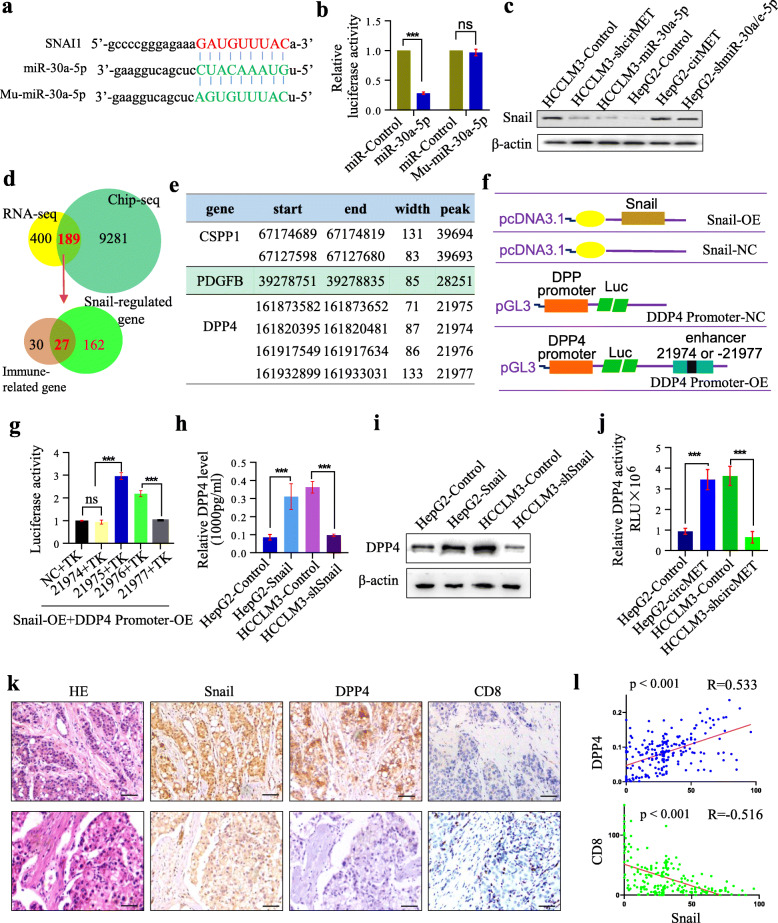
circMET induces immunosuppression in HCC through the miR-30-5p/Snail/DPP4 axis **a**. A schematic drawing showing the putative binding sites of human Snail and miR-30a-5p; **b**. The luciferase activity of luc-Snail in HEK293T cells after cotransfection with miR-30a-5p; **c**. The expression of Snail in HCC cells with different circMET and miR-30-5p expression levels; **d**. The overlap of RNA-seq data from cells with different circMET levels and Snail Chip-seq data, followed by overlap with the immune related genes from RNA-seq; **e**. The binding site of Snail with the genes CSPP1, PDGFB and DPP4 according to the Snail Chip-seq results; **f**. Diagrammatic sketch of the Snail-NC, Snail-OE, DPP4-promoter-Luc-NC and DPP4-promoter-Luc-OE vectors; **g**. The luciferase activity of the DPP4 reporter was upregulated in cells cotransfected with Snail DPP4-promoter and 21,975 + TK/21976 + TK; **h**. The DPP4 mRNA level is related to the level of Snail in HCC cells; **i**. The DPP4 protein level is related to the level of Snail in HCC cells; **j**. DPP4 levels in the supernatant of HCC cells with different levels of circMET; **k** and **l.** A positive relationship between Snail and DPP4, and a negative relationship between DPP4 and CD8^+^ T cells were observed in HCC tissues

We further determined the downstream mechanism of miR-30-5p in promoting HCC development. Based on the results of RNA-seq and an online prediction analysis, we speculated that Snail might be a target of miR-30-5p in humans. We first tested the putative binding sites of Snail with miR-30a-5p (Fig. 5a). A dual-luciferase reporter assay further showed that luciferase activity was reduced in cells transfected with wt-miR-30a-5p compared to cells transfected with mutant-miR-30a-5p (Fig. 5b).

To further elucidate the miR-30-5p functions in circMET-induced signaling, we constructed lentiviral vectors expressing miR-30a-5p, shmiR-30a/e-5p siRNA-miR-30b-5p, siRNA-miR-30c-5p, or siRNA-miR-30d-5p, and established stable cell lines. Snail expression was upregulated in HepG2-circMET, HepG2-shmiR-30a/e-5p, HepG2-simiR-30b-5p, HepG2-simiR-30c-5p, and HepG2-simiR-30d-5p cells compared to HepG2-contorl cells (Fig. 5c and Additional files 5**:** Fig. S2a, b), but was downregulated in HCCLM3-shcirMET and HCCLM3-miR-30a-5p compared to control cells (Fig. 5c**)**. These results indicate that snail is a target of miR-30-5p and that circMET could promote HCC progression via miR-30-5p/Snail.

Our data showed that circMET plays an important role in EMT and the microenvironment characterized by compromised immunosuppression, although the detailed mechanism is unknown. Considering that Snail acts as a transcription factor, we performed ChIP-seq to define the repertoire of Snail-binding sites in HepG2-circMET cells (Additional files 5**:**Fig. S2c). We identified a total of 42,479 high-confidence Snail-binding sites in 9470 genes in these cells (Additional files 3**:** Table S3). Binding sites (2.22, 2.17 and 1.94%) were found in promoter regions of genes (distances ≤1 kb, 1–2 kb and 2–3 kb from transcription start sites, TSS, respectively), 34% of sites were found in intron regions of genes, and 56.72% of sites were found in distal intergenic regions of genes (Additional files 5: Fig. S2d).

By overlapping differentially expressed genes between 9470 genes containing Snail-binding sites and the 589 differentially expressed genes between HepG2-control and HepG2-circMET cells, 189 genes were obtained, which were subjected to GO terms analysis for the immune-related genes, and finally 27 genes were identified as immune-related genes and potentially regulated by snail (Fig. 5d). In combination with the expression data of these genes in a larger cohort of HCC samples from The Cancer Genome Atlas (TCGA) and RNA-seq data (Additional files 5: Fig. S3), we further narrowed down Snail-regulated genes by excluding *COL1A1, COLEC11, CXCL12,* and *MBL2*, and found that *DPP4, PDGFB* and *CSPP1* might be candidate genes involved in snail-mediated immunosuppression (Fig. 5e), which was further tested by the dual-luciferase reporter assay. We found that Snail significantly upregulated DPP4 expression by interacting with the enhancer element of DPP4, while the two other genes were not directly or slightly influenced by Snail (Fig. 5f**,** Additional files 5: Fig. S4a-c). Dual-luciferase assays showed that snail interacted with distant 140,810, and 96,828 of DPP4 as enhancer elements to upregulate DPP4 expression (Fig. 5f, g and Additional files 5: Fig. S4d).

Additionally, we further verified that Snail upregulated DPP4 expression (Fig. 5h, i and j). We also identified a positive relationship between Snail and DPP4, and a negative relationship between Snail and CD8^+^ T cells in HCC tissues (Fig. 5k, l). Thus, we conclude that circMET overexpression induced a compromised immune microenvironment in HCC via the miR-30-5p/Snail/DPP4 axis.

### The miR-30-5p/snail/DPP4 axis induces immunosuppression by degrading CXCL10

**Fig. 6:**
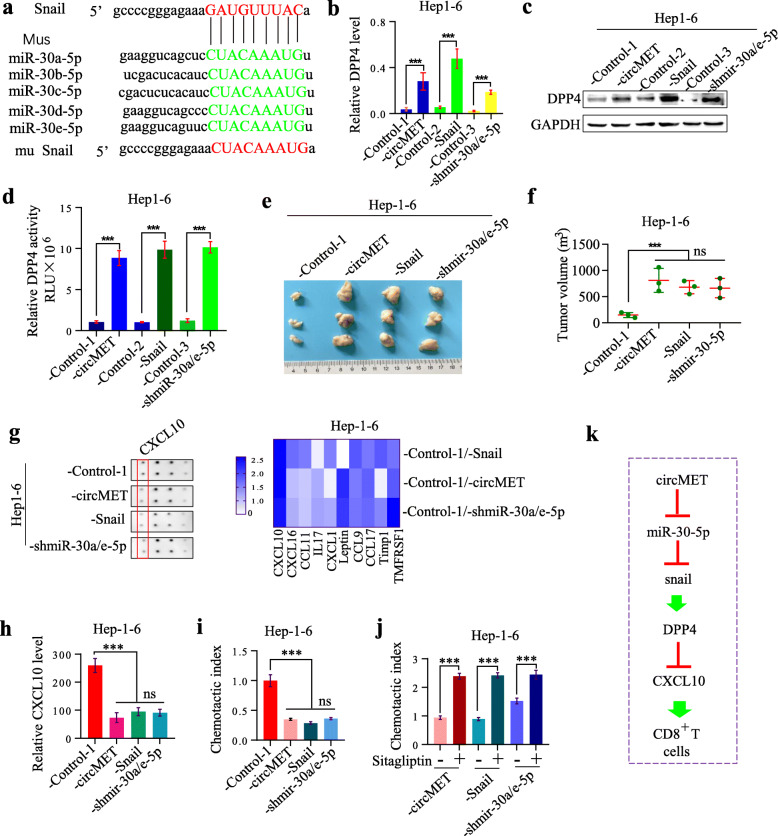
The miR-30-5p/Snail/DPP4 axis induces immunosuppression by degrading CXCL10 **a**. A schematic drawing showing the putative binding sites of human Snail and miR-30-5p and showing mutant Snail; **b**. DPP4 mRNA levels in Hep1–6 cells with different circMET, Snail and miR-30-5p expression; **c**. DPP4 protein levels in Hep1–6 cells with different circMET, Snail and miR-30-5p expression; **d**. DPP4 levels in the supernatant of Hep1–6 cells with different circMET, Snail and miR-30a-5p expression; control-1, control-2 and control-3 showed no differences, and control-1 was subsequently used as the control; **e**. The tumor volume of Hep1–6-control, Hep1–6-circMET, Hep1–6-Snail and Hep1–6-shmiR-30a/e-5p cells in C57BL/6 mice; **f**. The histogram shows the tumor volume of Hep1–6-control, Hep1–6-circMET, Hep1–6-Snail and Hep1–6-shmiR-30a/e-5p cells in C57BL/6 mice; **g**. Chemokine chips were used to determine the differences in chemokines between the sera of mice implanted with Hep1–6-control, Hep1–6-circMET, Hep1–6-Snail or Hep1–6-shmiR-30a/e-5p cells. Left representative pictures of chemokine chips. Right, statistic of chemokine chips; **h**. CXCL10 was further assessed by ELISA in cells with different expression levels of circMET, Snail and miR-30a/e-5p; **i**. The chemotactic index was detected in cells with different expression levels of circMET, Snail and miR-30a/e-5p; **j**. The influence of sitagliptin on the chemotactic index was detected in cells with different expression levels of circMET, Snail and miR-30a/e-5p; **k**. The novel mechanism by which circMET overexpression induces HCC progression

Previous studies have identified DPP4 truncated chemokines and other immune molecules that initiate their catabolism and clearance by other N­terminal aminopeptidases [33]. Therefore, we investigated the substrate of DPP4 and further revealed the mechanism of the miR-30-5p/Snail/DPP4 axis in HCC progression by using the DPP4 inhibitor, sitagliptin. We first showed that Snail was also the target of mouse mutant-miR-30-5p (mouse) (Fig. 6a), and that DPP4 was upregulated by overexpression of circMET and Snail or by interference with miR-30a/e-5p, miR-30b-5p, miR-30c-5p or miR-30d-5p in Hep1–6 cells (Fig. 6b-d**,** Additional file 5: Fig. S5a). The tumor volume in Hep1–6-circMET, −snail and -shmiR-30a/e-5p cells was much larger than that in the Hep1–6-control group (Fig. 6e, f).

Liquid Chip analysis showed that mouse serum CXCL10 (mCXCL10) levels were lower in mice implanted with Hep1–6-circMET, −snail, or -shmiR-30a/e-5p cells than in the controls (Fig. 6g**,** Additional file 5: Fig. S5b). These results were further verified by ELISA (Fig. 6h). Finally, the chemotactic index suggested that CXCL10 played an important role in CD8^+^ lymphocyte trafficking (Fig. 6i). Next, we directly assessed whether the DPP4 inhibitor sitagliptin increased CD8^+^ lymphocyte trafficking, and the chemotactic index suggested that DPP4 inhibition plays a role (Fig. 6j). Thus, we further confirmed that circMET influenced the immune response process in HCC via the miR-30-5p/Snail/DPP4 axis (Fig. 6k).

### Sitagliptin improves the response to anti-PD1 immunotherapy in a subgroup of HCC patients

**Fig. 7:**
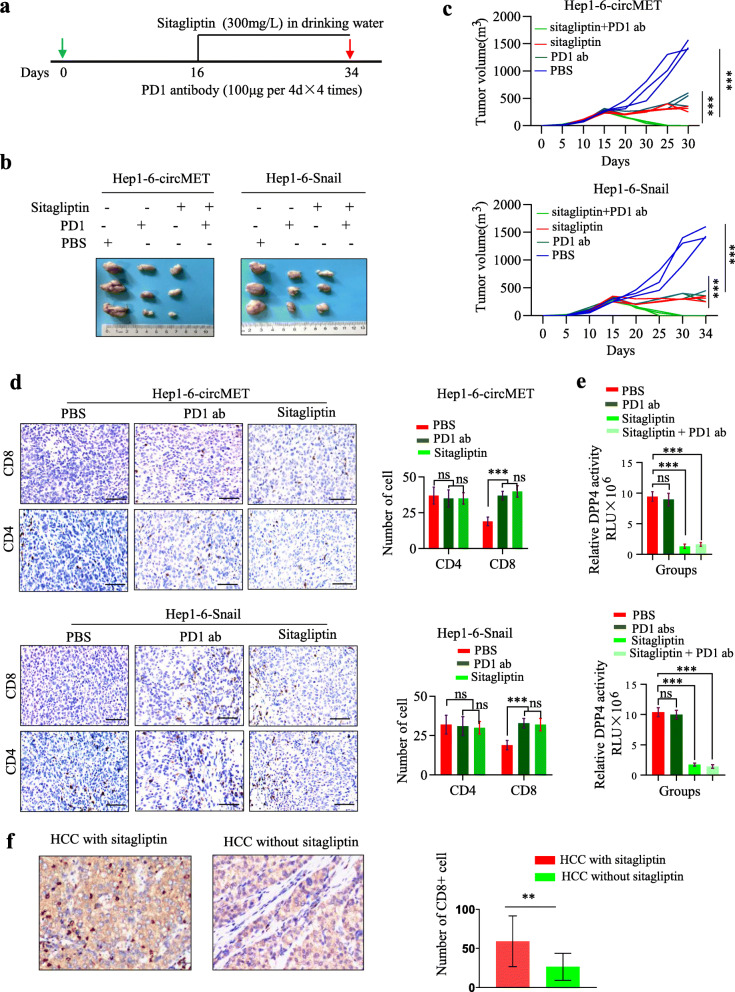
Sitagliptin improves the response to anti-PD1 immunotherapy in a subgroup of HCC **a**. Schematic of sitagliptin and anti-PD1 treatments; **b**. Representative images of Hep1–6 tumors from each group; **c**. Tumor growth curves of Hep-1-6 tumors from each group; **d**. CD4 and CD8 immunohistochemistry staining of tumors treated with PBS, sitagliptin or anti-PD1 antibody. Left, representative pictures of IHC staining. Right, statistic of CD4^+^ and CD8^+^ T cells per section (*n* = 3); **e**. DPP4 activities in tumors treated with PBS, sitagliptin or/and anti-PD1 antibody; **f**. CD8 immunohistochemistry staining of HCC from patients with diabetes treated with or without sitagliptin. Left, representative pictures of CD8 IHC staining. Right, statistic of CD8^+^ T cells per patient

DPP4 belongs to a family of six multidirectional serine proteases that cleave two amino acids at the N-termini of proteins and peptides [34]. The first oral incretin enhancer, sitagliptin, a selective DPP4 inhibitor, that has been approved for diabetes therapy. Combination therapy with anti-PD1 blockade immunotherapy and other therapies has been validated to improve the efficacy and duration of the tumor-specific T-cell response. Based on the above data related to sitagliptin, we predicted that sitagliptin could enhance lymphocyte trafficking and improve tumor responses to PD1 blockage in the clinical setting of HCC. Thus, we investigated the combination therapy of sitagliptin and PD1 inhibitor in the Hep1–6-circMET and Hep1–6-snail tumor model **(**Fig. 7a). Sitagliptin or anti-PD1 antibody monotherapy resulted in delayed Hep1–6-Snail and Hep1–6-circMET tumor growth compared to controls. Importantly, 100% tumor regression was observed in the group treated with sitagliptin plus anti-PD1 **(**Fig. 7b, c**;** Additional File 5: Fig. S5c, d).

A significant increase in the number of CD8^+^ T cells was found in the tumors of mice treated with sitagliptin (Fig. 7d). The serum DPP4 activity was inhibited in sitagliptin­treated mice and the mice treated with anti-PD1 antibodies combined with sitagliptin (Fig. 7e). Additionally, no significant differences were observed in the numbers of tumor-infiltrating myeloid, NK or B lymphocyte cells (data not shown).

We performed a retrospective cohort study to determine whether sitagliptin enhances CD8^+^ T lymphocyte trafficking in tumors. This study included 8 HCC patients with diabetes treated with sitagliptin between January 2018 and July 2019, and a control group of 32 HCC patients (1:4 matched) with diabetes who were not treated with sitagliptin. The results demonstrated that HCC tissues from patients who received sitagliptin showed higher CD8^+^ T cell infiltration, while HCC tissues from patients who did not receive sitagliptin showed significantly lower CD8 T cell infiltration (Fig. 7f), indicating that sitagliptin could enhance lymphocyte trafficking. Thus, the above results further indicated the circMET/miR-30-5p/Snail/DPP4 axis is involved in the immunosuppression of HCC, and suggest that sitagliptin could improve the efficacy of PD1 blockade immunotherapy.

## Discussion

A large number of circRNAs have been successfully identified in diverse cells and tissues owing to advances in high-throughput deep sequencing [35, 36]. Moreover, many circRNAs are expressed in a cell type-specific or tissue-specific manner, indicating that they might perform important biological functions [16]. Dysregulation of circRNAs was recently shown to be involved in several pathological processes, including neurological disorders, cardiac hypertrophy and cancer [36, 37]. By analyzing the expression of circRNAs in the 7q21-7q31 region, which was recently discovered to be a novel locus associated with both susceptibility to and prognosis of HBV-related HCC [12], we found that circMET (hsa_circ_0082002) expression was upregulated in HCC tissues compared to paratumor tissues, and that high levels of circMET were related to poor prognosis of HCC patients.

Moreover, we found that high levels of circMET induce cell EMT and are associated with a tumor suppressive microenvironment in HCC. Chip-seq and luciferase reporter assays showed that circMET overexpression induced an immunosuppressive tumor microenvironment via the miR-30-5p/Snail/ DPP4/CXCL10 axis. Importantly, we demonstrated that the DPP4 inhibitor sitagliptin significantly improved the antitumor effect of PD1 in immunocompetent mice bearing tumors with high levels of circMET and Snail. Clinically, we determined that sitagliptin may enhance CD8^+^ T cell infiltration in HCC patients. Thus, we revealed that circMET is a new onco-circRNA that induces HCC development and immune tolerance via the Snail/ DPP4/CXCL10 axis. DPP4 inhibitors thus may be able to enhance the efficacy of checkpoint-inhibitor therapy in a subgroup of HCC patients.

Amplification of chromosome 7q21-7q31 is implicated in cancer susceptibility, recurrence and multidrug resistance, including in HCC [12, 13]. Over the past decades, several oncogenes in this region, including *MET, HGF*, *SHFM1* and *PEG10,* have been revealed. Generally, a chromosomal aberration frequently harbors many cancer driver genes. According to previous reports [28], we assessed the expression of several circRNAs in this region in HCC and corresponding paratumor tissues. circMET was significantly upregulated in HCC tissues and HCC cells with high metastatic potential. Moreover, circMET overexpression induced HCC cell EMT and shaped the tumor suppressive microenvironment. Thus, we provide evidence that circMET is another oncogene in the chromosome 7q21-7q31 region.

Our data provide direct evidence reinforcing the notion that EMT is associated with tumor immune inhibition, whereby Snail could serves as a transcription factor of DPP4, which induces local immunosuppression by negatively regulating lymphocyte trafficking via cleavage of the chemokine CXCL10 [33]. As a Th1-type chemokine, CXCL10 plays an important role in determining effector T cell trafficking to the tumor microenvironment. Moreover, elevated CXCL10 in tumor cells can elicit potent tumor immunity, block cancer progression and enhance the clinical efficacy of immunotherapy [38]. Our results showed that tumors with circMET overexpression had low levels of CXCL10 and less CD8^+^ T cell infiltration than the control tumors. Moreover, we also showed a positive relationship between snail and DPP4, and a negative relationship between snail and CD8^+^ T cells in human HCC tissues. The above results indicated that circMET and Snail were associated with the tumor immunosuppressive microenvironment. Importantly, we further determined that the administration of sitagliptin, a DPP4 inhibitor, could act synergistically with anti-PD1 treatment in immunocompetent mice; simultaneously, CXCL10 was upregulated by the sitagliptin administration. Thus, we revealed that the circMET/snail/ DPP4/CXCL10 axis is a vital mechanism in HCC immunosuppression, and identified a subgroup of patients who can benefit from sitagliptin administration. This suggests a role for DPP inhibitors in improving immunotherapy in a subgroup of HCC patients, which was consistent with previous publications in melanoma [39]. Indeed, combined with the results of a retrospective cohort study conducted in HCC patients, we found that tissues from HCC patients with diabetes undergoing sitagliptin treatment had a higher level of CD8^+^ T lymphocyte infiltration than tissues from HCC patients with diabetes without sitagliptin treatment, which further supported that sitagliptin could elevate the efficacy of anti-PD1 antibody therapy.

Generally, changes in the expression of circRNAs are associated with linear transcripts derived from the same gene, but many genes exhibit differentially regulated circRNAs and linear RNA variants. Here, we found that MET expression had no relationship with circMET expression. Interestingly, RNA-seq results indicated that *HGF*, *SHFM1,* and *PEG10* were slightly downregulated in HepG2-circMET cells compared with HepG2-control cells, which we speculate may indicate a feedback inhibitory mechanism of circMET overexpression. Additionally, we also found that *MET* expression did not differ between cells with different circMET expression. Thus, the role of circMET in HCC progression is independent of MET function, which further supports the notion that circMET is a driver of HCC.

## Conclusions

In this study, we show that circMET acts as an onco-circRNA, and that circMET overexpression induces HCC development and immune tolerance via the miR-30-5p/Snail/ DPP4/CXCL10 axis. Furthermore, our results suggest that a DPP4 inhibitor could enhance the efficacy of PD1 inhibitor therapy in a subgroup of HCC patients.

### Supplementary information

**Additional file 1: Table S1.** The targets of sh/si-miR-30-5p.

**Additional file 2: Table S2.** The expressed genes in HepG2-circMET and HepG2-Mock.

**Additional file 3: Table S3.** Snail-binding sites in the snail CHIp-seq.

**Additional file 4:** Supplementary materials and methods

**Additional file 5: Figure S1.** a. The agreement between RNA-seq and qRT-PCR results; b. A negative relationship between CD8 and circMET; c. The relationship between CD4+ and CD8+ T cells in HCC tissues and the overall survival and cumulative recurrence of HCC patients was analyzed in 209 HCC patients.** Figure S2.** a. The expression of miR-30a/b/c/d/e-5p in HepG2 and HCCLM3 cells was determined by qRT-PCR; b. The expression of snail in HCC cells interfered with miR-30a/b/c/d/e-5p, (here we only showed miR-30b/c/d -5p); c. The chip peaks over chromosomes in snail Chip-seq; d The repertoire of snail-binding sites in HCC cell lines; **Figure S3.** The expression of 27 genes from the TCGA. **Figure S4.** a. The luciferase activity of DPP4 was upregulated in cells cotransfected with the Snail DDP4-promoter and 21,975 + TK/ 21,976 + TK; b. The luciferase activity of PDGFB after cotransfection of Snail and PDGFB vectors; c. The luciferase activity of CSPP1 after cotransfection of Snail and CSPP1 vectors; d. Snail interacts with distant sites 140,810 and 96,828 of DPP4 as enhancer elements to upregulate the DPP4 expression. **Figure S5.** a. The DPP4 level in Hep1–6 cells interfered with si-miR-30b-5p or si-miR-30c-5p or si-miR-30d-5p; b. Chemokine chips were used to determine the differences in chemokines between the sera of mice implanted with Hep-1-6-snail and the sera of mice implanted with Hep-1-6-shmiR-30s cells; c. Images of Hep1–6 tumors from each group and Tumor growth curves of Hep-1-6 tumors from each group(*n* = 3); d. Images of Hep1–6-circMET and Hep1–6-snail tumors from each group and Tumor growth curves of Hep-1-6-circMET and Hep1–6-snail tumors from each group (*n* = 3).

## Data Availability

All data generated or analyzed during this study are included either in this article or in the supplementary Materials and Methods, Tables, Figures and Figure Legends files.

## Abbreviations

CircRNA: Circular RNA;
HCC: Hepatocellular carcinoma;
miRNA: MicroRNA
PD01: Programmed cell death 1;
MET: MET proto-oncogene receptor tyrosine kinase
circRIP: CircRNA immunoprecipitation
qRT-PCR: Quantitative real-time polymerase chain reaction
IHC: Immunohistochemistry;
CHIP-seq: Chromatin immunoprecipitation sequencing
DPP4: Dipeptidyl peptidase 4
