# Correction: Circular RNA circMET drives immunosuppression and anti-PD1 therapy resistance in hepatocellular carcinoma via the miR-30-5p/snail/DPP4 axis

**DOI:** 10.1186/s12943-024-01969-1

**Published:** 2024-02-29

**Authors:** Xiao-Yong Huang, Peng-Fei Zhang, Chuan-Yuan Wei, Rui Peng, Jia-Cheng Lu, Chao Gao, Jia-Bing Cai, Xuan Yang, Jia Fan, Ai-Wu Ke, Jian Zhou, Guo-Ming Shi

**Affiliations:** 1 Department of Liver Surgery and Transplantation, Liver Cancer Institute, Zhongshan Hospital, Fudan University, Key Laboratory of Carcinogenesis and Cancer Invasion, Ministry of Education, Fudan University, 180 Fenglin Road, Shanghai, 200032 P.R. China; 2 Cancer Center, Institutes of Biomedical Sciences, Fudan University, Shanghai, 200031 P.R. China; 3 State Key Laboratory of Genetic Engineering and Collaborative Innovation Center for Genetics and Development, School of Life Sciences, Fudan University, Shanghai, China; 4 Shanghai Key Laboratory of Organ Transplantation, Zhongshan Hospital, Fudan University, Shanghai, China

**Correction** **:** **Mol Cancer 19, 92 (2020)**

**https://doi.org/10.1186/s12943-020-01213-6**

Following publication of the original article [1], the authors would like to correct an error in Fig. 7d (CD4 and CD8 staining in Hep1-6-Snail tumors treated by PBS, PD1 abs and Sitagliptin. The other Figures of the article remain the same, and the interpretation of the results remains unchanged. The correction does not affect the conclusion or discussion of this article. The correct and incorrect figures are given below.

Incorrect Fig. 7:


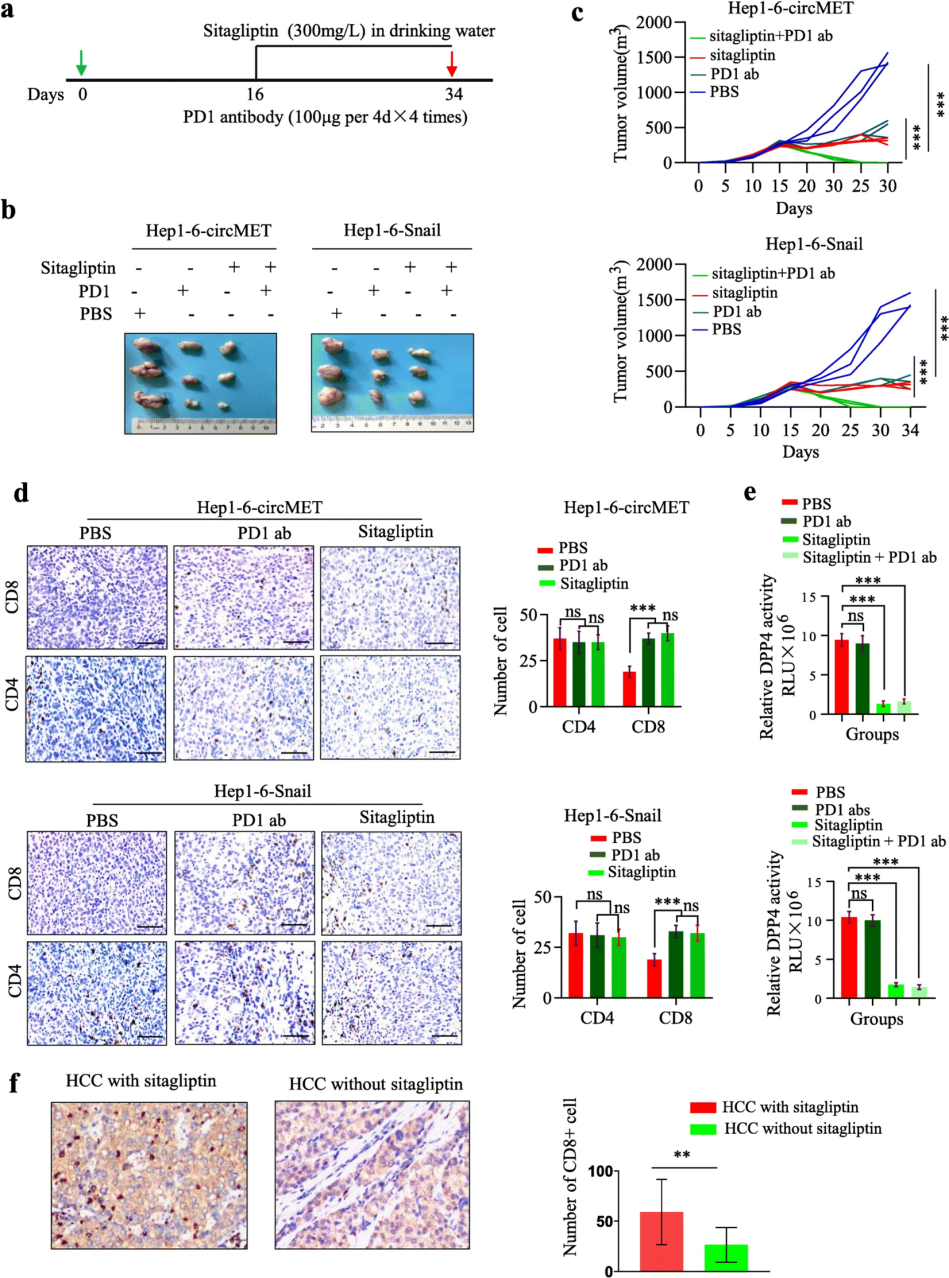


Correct Fig. 7:

**Fig. 7:**
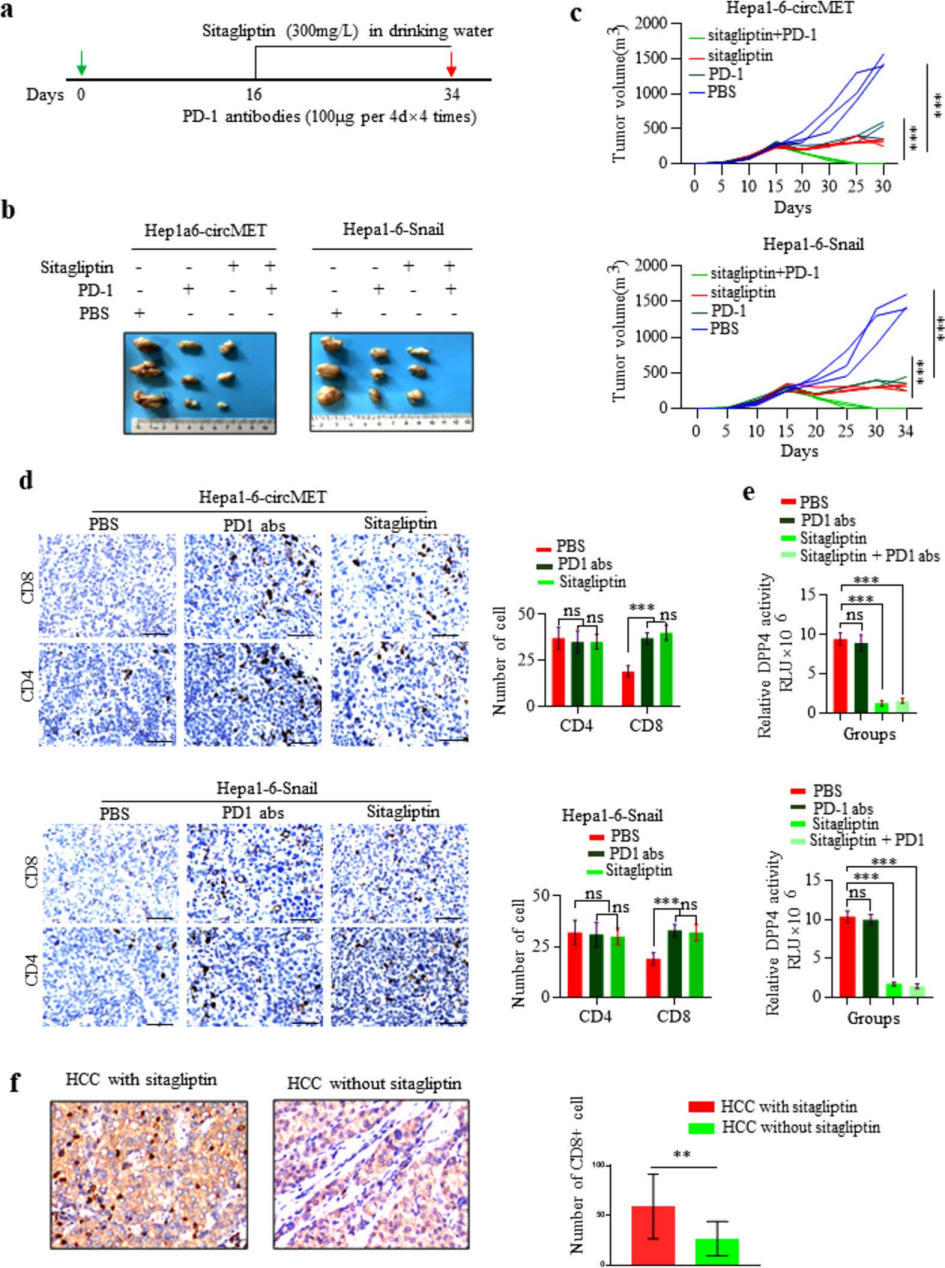
Sitagliptin improves the response to anti-PD1 immunotherapy in a subgroup of HCC **a**. Schematic of sitagliptin and anti-PD1 treatments; **b**. Representative images of Hep1–6 tumors from each group; **c**. Tumor growth curves of Hep-1–6 tumors from each group; **d**. CD4 and CD8 immunohistochemistry staining of tumors treated with PBS, sitagliptin or anti-PD1 antibody. Left, representative pictures of IHC staining. Right, statistic of CD4^+^ and CD8^+^ T cells per section (*n* = 3); **e**. DPP4 activities in tumors treated with PBS, sitagliptin or/and anti-PD1 antibody; **f**. CD8 immunohistochemistry staining of HCC from patients with diabetes treated with or without sitagliptin. Left, representative pictures of CD8 IHC staining. Right, statistic of CD8^+^ T cells per patient.
