# Supplementary material for: Circular RNA circMET drives immunosuppression and anti-PD1 therapy resistance in hepatocellular carcinoma via the miR-30-5p/snail/DPP4 axis
Source: Mol Cancer. 2020 May 19;19:92. doi: 10.1186/s12943-020-01213-6 (PMC7236145; doi:10.1186/s12943-020-01213-6)
Supplement: Supplementary file 1 — Additional file 1: Table S1. The targets of sh/si-miR-30-5p. [file 12943_2020_1213_MOESM1_ESM.doc]

**The targets of sh/si-miR-30-5p**:

| miR-30a-5p and 30e-5p shRNA target | UGUAAACAUCCUACACUC |
| --- | --- |
| miR-30b-5p siRNA targe | UACAUGGAUUGGCUGGGA |
| miR-30c-5p siRNA target | CAUCCUACACUCUCAGCUG |
| miR-30d-5p siRNA target | GACACAGCUAAGCUUUCAG |
