# Supplementary material for: Circular RNA circMET drives immunosuppression and anti-PD1 therapy resistance in hepatocellular carcinoma via the miR-30-5p/snail/DPP4 axis
Source: Mol Cancer. 2020 May 19;19:92. doi: 10.1186/s12943-020-01213-6 (PMC7236145; doi:10.1186/s12943-020-01213-6)
Supplement: Supplementary file 4 — Additional file 4: Supplementary materials and methods [file 12943_2020_1213_MOESM4_ESM.docx]

**RNA expression analysis, western blot analysis, immunofluorescence assay and confocal immunofluorescence.**

RNA expression was analyzed by using the ABI PRISM 7900 Sequence Detection System (Applied Biosystems, Foster City, CA, USA) starting with 1 µl cDNA and SYBR Green Real-time PCR Master Mix. Each sample was tested in triplicate.

Protein extracts from the cells were electrophoresed by sodium dodecyl sulfate-polyacrylamide gel electrophoresis (SDS-PAGE), and then transferred onto polyvinylidene difluoride membranes, which were incubated with the corresponding antibodies overnight. The enhanced chemiluminescence method was used to detect the expression levels.

The cells were permeabilized with 0.1% Triton X-100 for 15 min at room temperature, and then blocked with PBS containing 5% bovine serum albumin (BSA) for one hour. Cells were treated with the primary antibody overnight，washed with PBS and then incubated with secondary antibody. The slices were examined using fluorescence microscopy for confocal immunofluorescence (Leica Microsystems Imaging Solutions, Cambridge, UK).

**Cell Matrigel invasion assays and MTT assay**

Cell invasion assays were performed using a 24-well transwell (pore size 8 μm; Corning) precoated with Matrigel (Falcon 354480; BD Biosciences). A total of 1 x 10^5^ cells were then suspended in 500 μl of DMEM containing 1% FBS and added to the upper chamber, and 750 μl of DMEM containing 10% FBS was placed in the lower chamber. After 48 hours of incubation, the Matrigel and all remaining cells in the upper chamber were removed with a cotton swab. The cells on the lower surface of the membrane were fixed. The cells in the three microscope fields (magnification, 200 x) were counted and imaged. All experiments were repeated three times.

Cells were aliquoted into 96-well plates (2000 cells in 200 μl per well) and incubated in medium. Then 20 μl of 3-[4,5-dimethylthiazol-2-yl]-2,5-diphenyltetrazolium bromide (MTT) bromine solution was added at the indicated time points and the aliquots were incubated for 4 hours. The medium was replaced with 150 μl of dimethyl sulfoxide and the aliquot was shaken for 10 minutes. The absorbance at 560 nm was measured to determine the number of viable cells in each well. All experiments were repeated three times.

**circRNA immunoprecipitation (circRIP) and in situ hybridization**

RNA immunoprecipitation (RIP) assays were performed using a Magna RIP RNA Binding Protein Immunoprecipitation Kit (Millipore) according to the manufacturer’s instructions with a cirRNA-0082002 probe. An anti-Argonaute 2 (AGO2) and IgG antibodies were used in this study.

In situ hybridization was performed using specific probes directed to the circRNA-MET sequence. The FISH probe was as follows: AGGGCAATAATCCCAAGGAATGAGTGGATTTCCCGTGTA GC ACCAGGGTC.

**Total RNA isolation and RNA sequencing**

Total RNA was extracted from HCC cell lines using TRizol reagent (Invitrogen) and then purified with TRizol. Purified RNA was treated with RNase R (Epicenter, 40 U, 37°C, 3 h). The RNA-seq library was generated using the NEBNext Ultra RNA Library Preparation Kit and subjected deep sequencing with the Illumina HiSeq 3000 system.

**Chemokine chip**

A total of 100 μL of sample dilution buffer was added to each well and incubated for 1 h on a shaker at room temperature to block the quantitative antibody chip. The buffer in each well was removed, 100 μL of samples and standards were added to the wells, and the chip was incubated overnight at 4 °C. The Thermo Scientific Well Wash Versa chip washer was used to clean the slide. For the antibody mixture incubation, the antibody mixture tubes were centrifuged, 1.4 ml of the sample dilution was added, and the solution was mixed well and centrifuged again quickly. Eighty microliters of the detection antibody was added to each well and incubated on an RT shaker for 2 hours. For the Cy3-streptavidin incubation, the Cy3-streptavidin tube was centrifuged, 1.4 ml of the sample dilution was added, and the solution was mixed well and centrifuged again quickly. Eighty microliters of Cy3-streptavidin was added to each well, and the slides were wrapped in aluminum foil to protect the slides from light and incubated for 1 hour on an RT shaker. Fluorescence detection was performed.
