# Supplementary figures and images for: Circular RNA circMET drives immunosuppression and anti-PD1 therapy resistance in hepatocellular carcinoma via the miR-30-5p/snail/DPP4 axis
Source: Mol Cancer. 2020 May 19;19:92. doi: 10.1186/s12943-020-01213-6 (PMC7236145; doi:10.1186/s12943-020-01213-6)

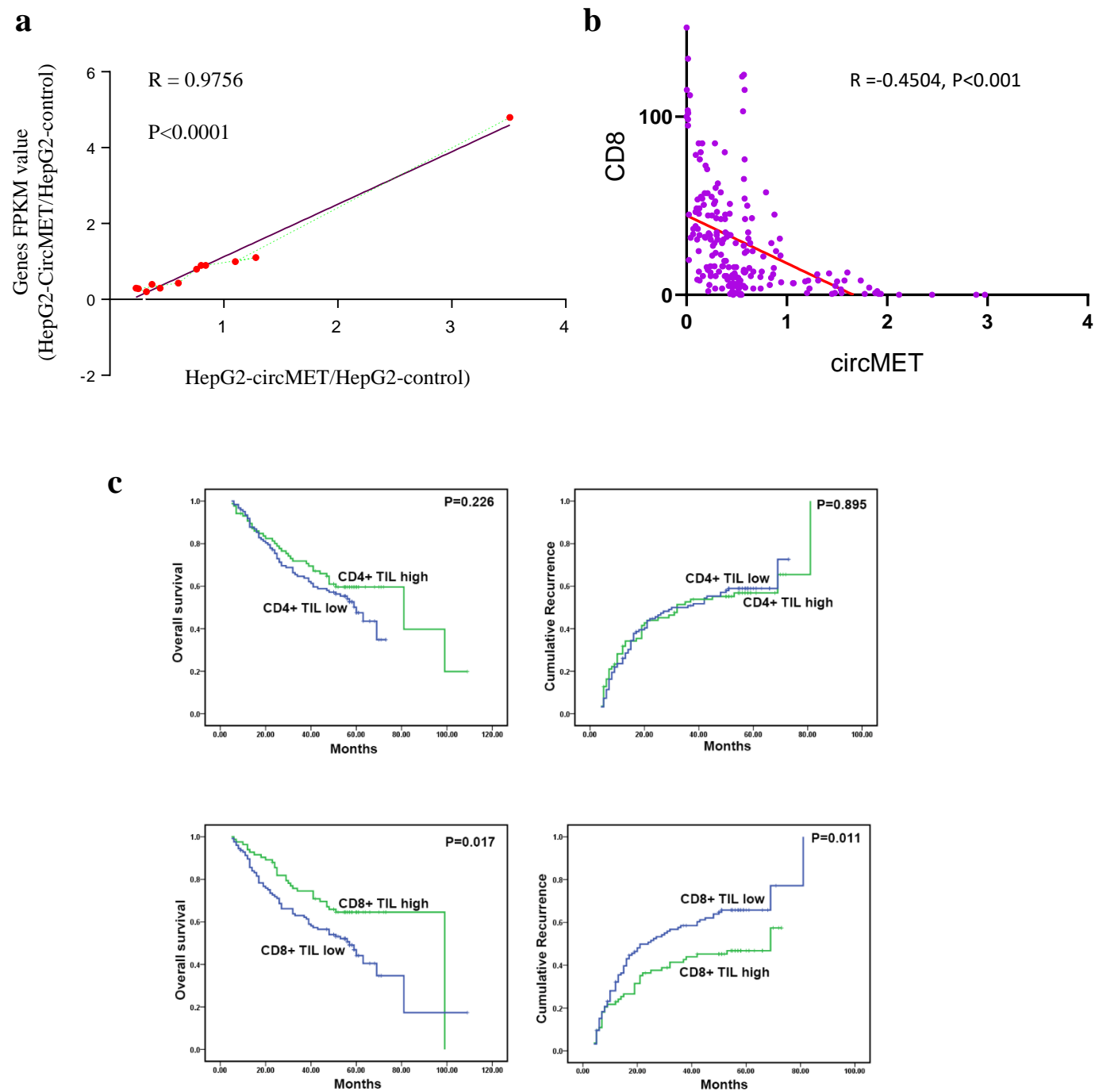

**Figure S1**

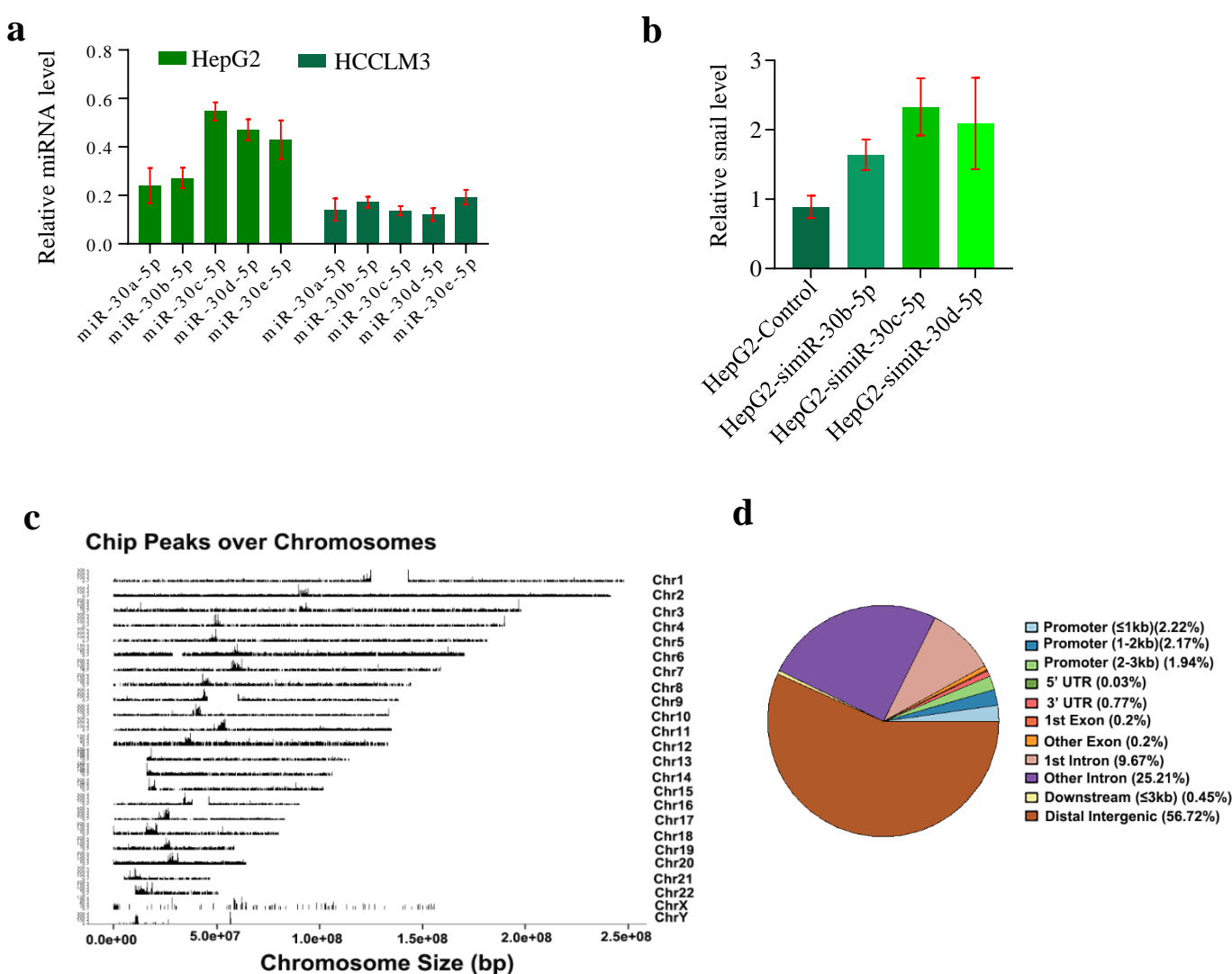

Figure S2

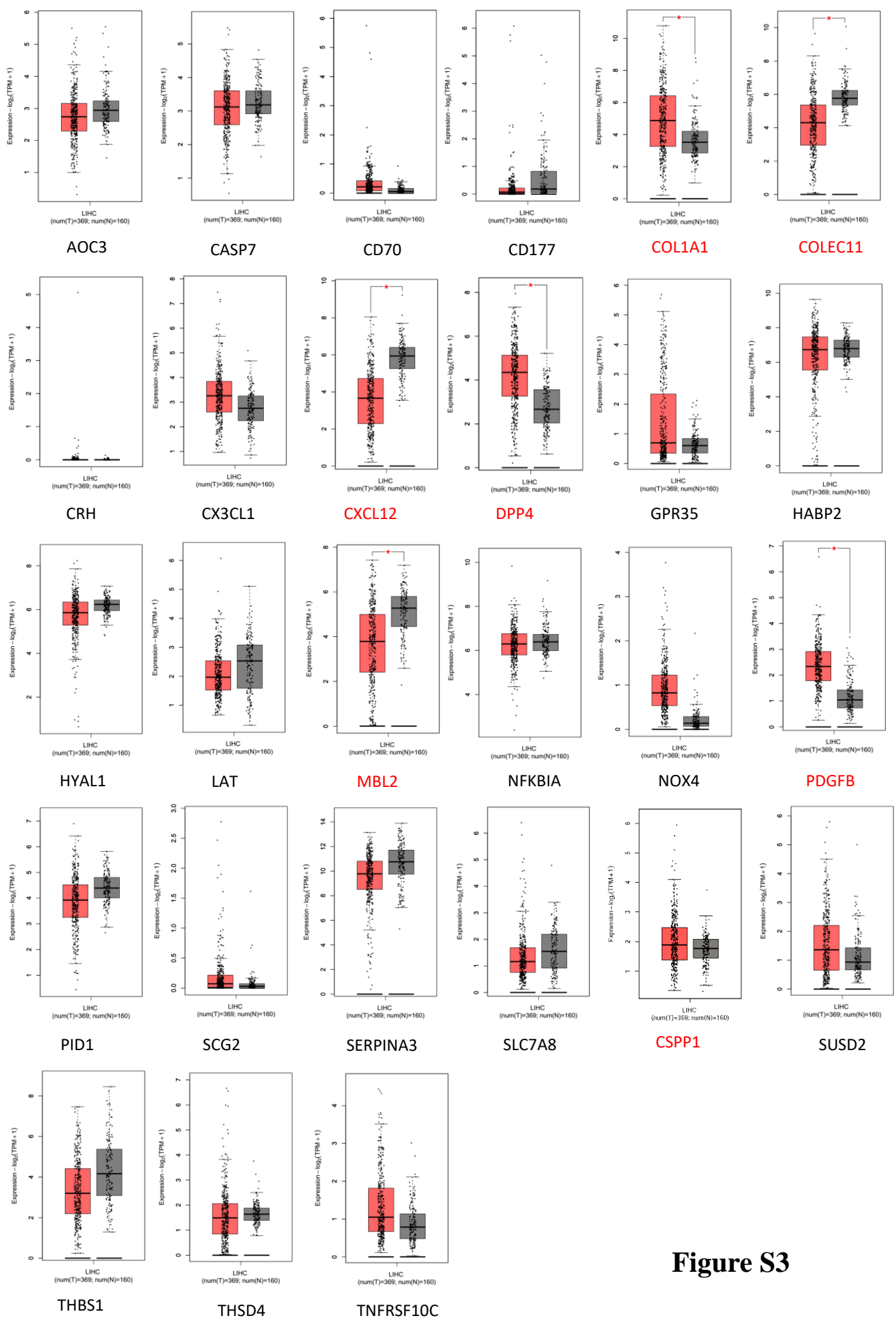

**Figure S3**

**a**

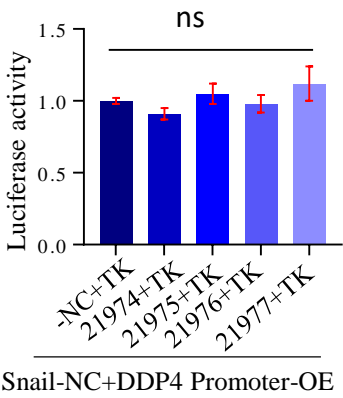

**b**

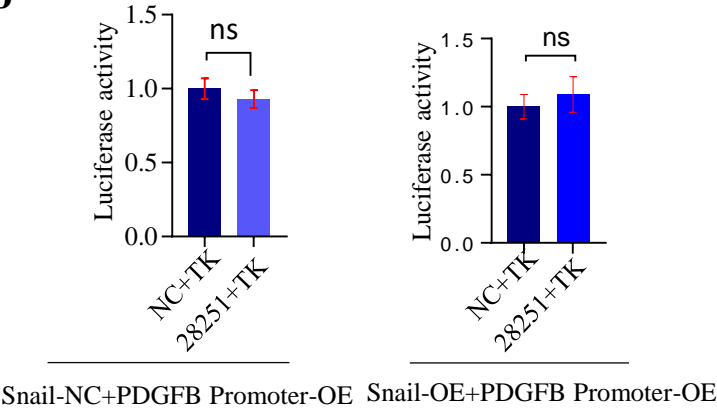

**c**

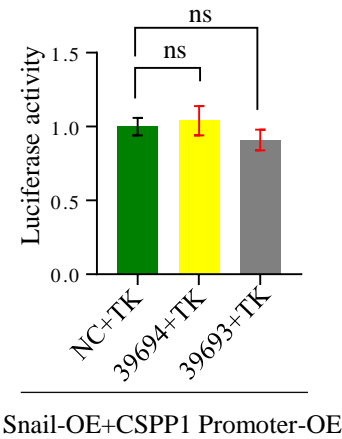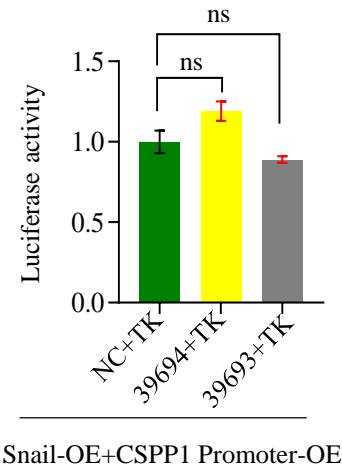

**d**

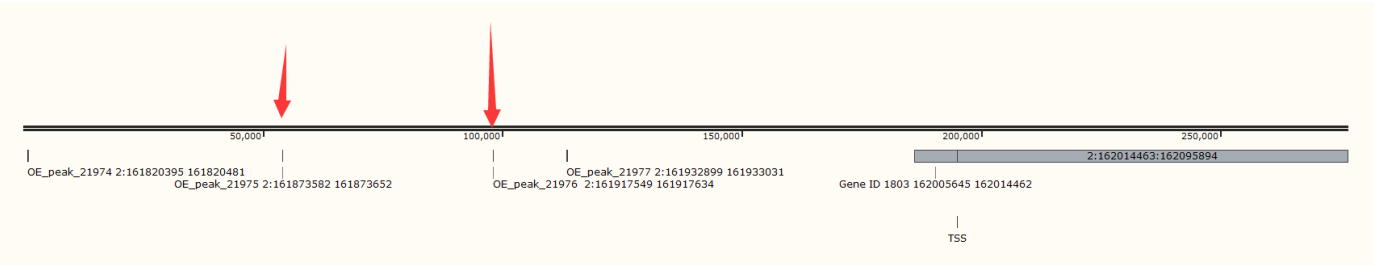

**Figure S4**

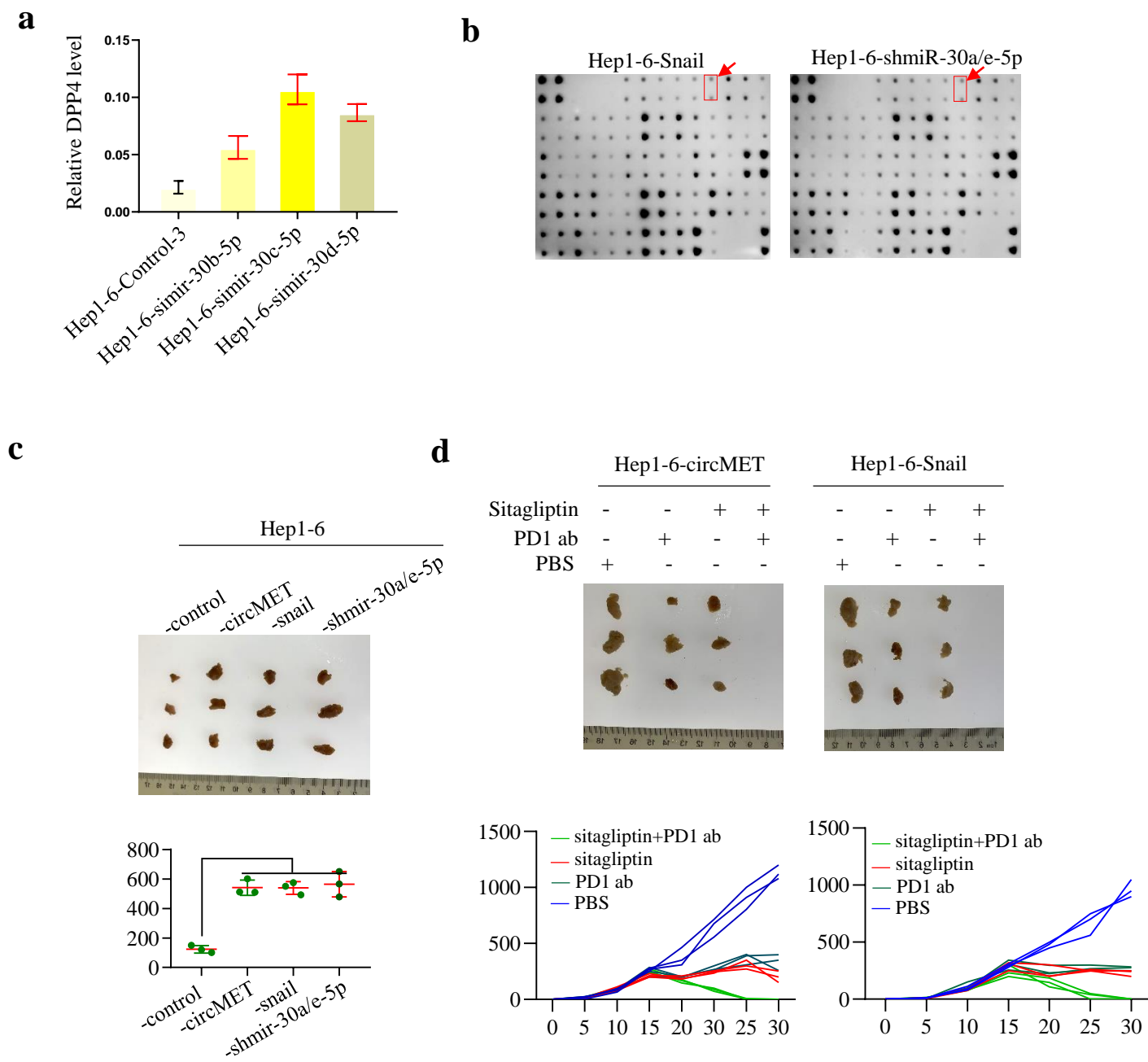

**Figure S5**

Supplement: Supplementary file 5 — Additional file 5: Figure S1. a. The agreement between RNA-seq and qRT-PCR results; b. A negative relationship between CD8 and circMET; c. The relationship between CD4+ and CD8+ T cells in HCC tissues and the overall survival and cumulative recurrence of HCC patients was analyzed in 209 HCC patients. Figure S2. a. The expression of miR-30a/b/c/d/e-5p in HepG2 and HCCLM3 cells was determined by qRT-PCR; b. The expression of snail in HCC cells interfered with miR-30a/b/c/d/e-5p, (here we only showed miR-30b/c/d -5p); c. The chip peaks over chromosomes in snail Chip-seq; d The repertoire of snail-binding sites in HCC cell lines; Figure S3. The expression of 27 genes from the TCGA. Figure S4. a. The luciferase activity of DPP4 was upregulated in cells cotransfected with the Snail DDP4-promoter and 21,975 + TK/ 21,976 + TK; b. The luciferase activity of PDGFB after cotransfection of Snail and PDGFB vectors; c. The luciferase activity of CSPP1 after cotransfection of Snail and CSPP1 vectors; d. Snail interacts with distant sites 140,810 and 96,828 of DPP4 as enhancer elements to upregulate the DPP4 expression. Figure S5. a. The DPP4 level in Hep1–6 cells interfered with si-miR-30b-5p or si-miR-30c-5p or si-miR-30d-5p; b. Chemokine chips were used to determine the differences in chemokines between the sera of mice implanted with Hep-1-6-snail and the sera of mice implanted with Hep-1-6-shmiR-30s cells; c. Images of Hep1–6 tumors from each group and Tumor growth curves of Hep-1-6 tumors from each group(n = 3); d. Images of Hep1–6-circMET and Hep1–6-snail tumors from each group and Tumor growth curves of Hep-1-6-circMET and Hep1–6-snail tumors from each group (n = 3). [file 12943_2020_1213_MOESM5_ESM.pdf]
